# Ribosomal DNA Sequence Heterogeneity Reflects Intraspecies Phylogenies and Predicts Genome Structure in Two Contrasting Yeast Species

**DOI:** 10.1093/sysbio/syu019

**Published:** 2014-03-27

**Authors:** Claire West, Stephen A. James, Robert P. Davey, Jo Dicks, Ian N. Roberts

**Affiliations:** ^1^National Collection of Yeast Cultures, Institute of Food Research, Norwich Research Park, Norwich NR4 7UA, UK; ^2^Bioinformatics, The Genome Analysis Centre, Norwich Research Park, Norwich NR4 7UH, UK; ^3^Department of Computational and Systems Biology, John Innes Centre, Norwich Research Park, Norwich NR4 7UH, UK

## Abstract

The ribosomal RNA encapsulates a wealth of evolutionary information, including genetic variation that can be used to discriminate between organisms at a wide range of taxonomic levels. For example, the prokaryotic 16S rDNA sequence is very widely used both in phylogenetic studies and as a marker in metagenomic surveys and the internal transcribed spacer region, frequently used in plant phylogenetics, is now recognized as a fungal DNA barcode. However, this widespread use does not escape criticism, principally due to issues such as difficulties in classification of paralogous versus orthologous rDNA units and intragenomic variation, both of which may be significant barriers to accurate phylogenetic inference. We recently analyzed data sets from the Saccharomyces Genome Resequencing Project, characterizing rDNA sequence variation within multiple strains of the baker's yeast *Saccharomyces cerevisiae* and its nearest wild relative *Saccharomyces paradoxus* in unprecedented detail. Notably, both species possess single locus rDNA systems. Here, we use these new variation datasets to assess whether a more detailed characterization of the rDNA locus can alleviate the second of these phylogenetic issues, sequence heterogeneity, while controlling for the first. We demonstrate that a strong phylogenetic signal exists within both datasets and illustrate how they can be used, with existing methodology, to estimate intraspecies phylogenies of yeast strains consistent with those derived from whole-genome approaches. We also describe the use of partial Single Nucleotide Polymorphisms, a type of sequence variation found only in repetitive genomic regions, in identifying key evolutionary features such as genome hybridization events and show their consistency with whole-genome Structure analyses. We conclude that our approach can transform rDNA sequence heterogeneity from a problem to a useful source of evolutionary information, enabling the estimation of highly accurate phylogenies of closely related organisms, and discuss how it could be extended to future studies of multilocus rDNA systems. [concerted evolution; genome hydridisation; phylogenetic analysis; ribosomal DNA; whole genome sequencing; yeast]

Ribosomal DNA (rDNA) is arranged at one or more loci in arrays of tandem elements. For example, in the baker's yeast *Saccharomyces cerevisiae*, approximately 150 rDNA units of 9137 bp in length are found in tandem at a single locus on chromosome XII, comprising approximately 1.4 Mb or ∼10% of the genome length. In contrast, humans possess ∼350 rDNA units of approximately 43 kb (including a large 30 kb intergenic spacer) organized in tandem clusters on the short arms of five acrocentric chromosomes, in this case ∼0.5% of the genome length. rDNA tandem arrays are believed to evolve via one or more concerted evolutionary mechanisms, such as unequal sister chromatid exchange and gene conversion, which both promote sequence homogeneity along the array (by either removing or amplifying novel variants) while potentially changing the number of elements within it (Eickbush and Eickbush 2007).

The ubiquity of rDNA, essential for protein synthesis, makes it a key target for evolutionary studies. rDNA sequences and subsequences are commonly used for determining species identity and inferring genetic interrelationship (Woese 2000). For example, in yeast the variable D1/D2 region of the large subunit (LSU) rRNA gene has proved an invaluable first step in species identification and is important in both molecular barcoding and phylogenetic reconstruction (Kurtzman and Robnett 1998; Fell et al. 2000). In plants, the internal transcribed spacer (ITS) has become widely used in phylogenetic inference (Álvarez and Wendel 2003). In prokaryotes, the 16S component of the small subunit (SSU) rRNA is widely used as a marker in metagenomics studies (Handelsman 2004). Furthermore, the ITS sequence has recently been proposed as the primary DNA barcode marker for *Fungi* (Schoch et al. 2012).

In addition to the universality of the rDNA sequence, other advantages of its use exist, such as biparental inheritance, ease of PCR amplification of subsequences such as the ITS, and intergenomic variability within both species and genus (Baldwin et al. 1995). However, several potential pitfalls in the use of rDNA for phylogenetic inference have been noted (Álvarez and Wendel 2003). These issues include difficulty in resolving paralogous from orthologous sequences (in cases of multilocus rDNA systems), incomplete intragenomic sequence homogeneity, the presence of rDNA pseudogenes, secondary structure considerations, difficulties in sequence alignment, frequent ITS sequence contamination, and homoplasy (Álvarez and Wendel 2003). Although it could be argued that the potential effects of some of these issues could be alleviated or lessened through alternative laboratory or analytical practises, others such as paralogy and sequence heterogeneity are more difficult to overcome. Indeed, sequence heterogeneity within the rDNA unit has long been a problem in phylogenetic analysis of many species groups, with numerous studies citing this issue, in particular within the ITS region (Buckler et al. 1997; Álvarez and Wendel 2003; Nilsson et al. 2008; Kiss 2012).

Using Whole Genome Shotgun Sequencing (WGSS) reads from the Saccharomyces Genome Resequencing Project (SGRP), we finely characterized rDNA sequence variation in multiple strains of *S. cerevisiae* for the first time (James et al. 2009), reporting high levels of sequence variation among individual rDNA units, ranging from 10 to 76 polymorphisms per strain across 227 variable sites. Many of the detected polymorphisms were not fully resolved across all units of the tandem array. For this type of intragenomic variation we introduced the term partial Single Nucleotide Polymorphism, or pSNP, as it is yet to become fixed by forces of concerted evolution. Furthermore, we showed an intriguing link between the number of pSNPs harbored by an individual strain and whether that strain was classified as possessing a *structured* genome, having arisen from a distinct lineage, or a *mosaic* genome, thought to have resulted from hybridization of divergent strains. More recently, we carried out a new analysis of rDNA sequence variation within *S. cerevisiae* and its wild relative *S. paradoxus* (West et al., in preparation), with our application of the TURNIP software (Davey et al. 2010) enabling the examination of a broader range of mutation types than in our earlier study.

Here, we attempt to derive accurate intraspecies phylogenies directly from the sequence variation datasets resulting from this recent study, thereby removing sequence heterogeneity as a phylogenetic problem. Crucially, the two species investigated both possess single-locus rDNA systems, so we effectively control for phylogenetic conflict derived from incorrect homology classification. We discover that by coding the extensive set of intra- and inter-genomic polymorphisms as allele frequency data, they may be used successfully for yeast intraspecies phylogenetic analysis. We refine our previous association of pSNP number and genome hybridization and apply it to our two strain sets, unexpectedly identifying putative hybrid strains in both species. Finally, we discuss the implications of our study for the phylogenetic analysis of multilocus rDNA systems.

## Methods

### Datasets

We recently analyzed raw WGSS reads for 26 *S. paradoxus* and 34 *S. cerevisiae* strains analyzed within the SGRP. In this analysis, the S288c *S. cerevisiae* and CBS432 *S. paradoxus* rDNA consensus sequences were used as templates against which to map polymorphisms in the remaining strains from the same species, using the TURNIP software (Davey et al. 2010). In total, we identified 778 and 654 SNP or pSNP polymorphisms in *S. paradoxus* and *S. cerevisiae*, respectively (Tables 1 and 2; West et al., in preparation). Here, we additionally scored variation between rDNA-specific reads of the *S. cerevisiae* type strain S288c and the *S. paradoxus* strain Q32.3 against the *S. paradoxus* and *S. cerevisiae* rDNA consensus sequences respectively, again using TURNIP (all parameters used were set to default values except for the BLAST parameters -b and -v, which were increased to 800 in order to allow all reads aligning to specific rDNA regions to be stored and analyzed). The *S. paradoxus* strain Q32.3 was used instead of the type strain CBS432 as the SGRP dataset for the latter was found in our earlier analysis to contain contaminated reads. Online Appendices 1 and 2 (available from http://dx.doi.org/10.5061/dryad.0674n) show the SNP and pSNP outputs for the *S. paradoxus* and *S. cerevisiae* datasets, respectively.

**Table 1.** rDNA sequence variation uncovered within the *S. paradoxus* dataset

Strain	Population	SNP	pSNP	Total	Copy Number (S.E.)
Q32.3	European	0	0	0	74 (0.109)
Q89.8	European	0	0	0	81 (0.228)
Q95.3	European	0	0	0	46 (0.093)
S36.7	European	0	0	0	57 (0.109)
T21.4	European	0	0	0	66 (0.074)
Y6.5	European	1	0	1	65 (0.085)
Y7.2	European	1	0	1	78 (0.108)
Z1.1	European	1	0	1	83 (0.103)
Q62.5	European	2	2	4	68 (0.096)
CBS 432 (T)	European	5	0	5	68 (0.072)
Q59.1	European	0	5	5	52 (0.062)
DBVPG 4650	European	2	4	6	87 (0.107)
KPN 3828	European	7	1	8	82 (0.112)
KPN 3829	European	7	1	8	79 (0.124)
CBS 5829	European	6	3	9	88 (0.095)
N-17	European	1	17	18	78 (0.068)
IFO 1804	Far Eastern	39	0	39	96 (0.187)
N-44	Far Eastern	38	1	39	52 (0.085)
N-45	Far Eastern	4	36	40	66 (0.049)
N-43	Far Eastern	40	1	41	64 (0.126)
A12	American	84	0	84	45 (0.065)
A4	American	88	0	88	66 (0.098)
UFRJ 50816	American	92	0	92	72 (0.090)
UFRJ 50791	American	95	0	95	64 (0.107)
YPS138	American	95	0	95	76 (0.099)
DBVPG 6304	American	97	2	99	53 (0.094)
Total		705	73	778	

Notes: Table of SNP and pSNP polymorphisms for each *S. paradoxus* strain, compared to the reference strain CBS432, as identified using the TURNIP software. Polymorphism counts are taken from West et al., in preparation. For each strain, the population and estimated ribosomal DNA copy number (along with the standard error of the copy number estimate) are also given. Ordering the strains by total polymorphism count results in the strains being split into their population groups.

**Table 2.** rDNA sequence variation uncovered within the *S. cerevisiae* dataset

Strain	Group	Genome type	Modified genome type	SNP	pSNP	Total	Copy number (S.E.)
W303	OM	Mosaic	Mosaic	0	3	3	182 (0.142)
L_1374	W/E	Structured	Structured mosaic	6	2	8	60 (0.096)
DBVPG 1106	W/E	Structured	Structured mosaic	7	1	8	98 (0.112)
DBVPG 1788	W/E	Structured	Structured mosaic	8	0	8	67 (0.101)
YJM981	W/E	Structured	Structured mosaic	6	3	9	354 (0.495)
YJM975	W/E	Structured	Structured mosaic	6	4	10	65 (0.095)
YJM978	W/E	Structured	Structured mosaic	6	4	10	65 (0.136)
YPS128	NA	Structured	Structured clean	14	0	14	62 (0.094)
S288c	OM	Mosaic	Mosaic	0	14	14	111 (0.163)
BC187	W/E	Structured	Structured mosaic	7	7	14	71 (0.135)
DBVPG 1373	W/E	Structured	Structured mosaic	8	7	15	75 (0.127)
DBVPG 6765	W/E	Structured	Structured mosaic	13	3	16	70 (0.077)
YPS606	NA	Structured	Structured clean	14	2	16	67 (0.096)
NCYC 110	WA +	Structured	Structured clean	15	2	17	163 (0.199)
DBVPG 6044	WA +	Structured	Structured clean	15	2	17	107 (0.121)
Y9	SA	Structured	Structured mosaic	8	10	18	79 (0.149)
UWOPS87-2421	UM	Mosaic	Mosaic	14	4	18	57 (0.109)
322134S	OM	Mosaic	Mosaic	6	12	18	109 (0.140)
SK1	WA +	Mosaic	Mosaic	16	3	19	72 (0.080)
27361N	OM	Mosaic	Mosaic	4	15	19	93 (0.119)
Y12	SA	Structured	Structured mosaic	9	11	20	78 (0.143)
378604X	OM	Mosaic	Mosaic	0	20	20	87 (0.117)
Y55	WA +	Mosaic	Mosaic	15	7	22	72 (0.060)
K11	SA	Structured	Structured mosaic	23	2	25	50 (0.082)
YIIc17_E5	YII	Mosaic	Mosaic	7	18	25	80 (0.117)
DBVPG 6040	OM	Mosaic	Mosaic	0	27	27	132 (0.106)
NCYC 361	OM	Mosaic	Mosaic	0	27	27	189 (0.189)
YS9	OM	Mosaic	Mosaic	1	27	28	56 (0.130)
UWOPS83-787-3	UM	Mosaic	Mosaic	8	21	29	64 (0.102)
UWOPS03-461-4	MA	Structured	Structured clean	29	0	29	89 (0.090)
UWOPS05-217-3	MA	Structured	Structured clean	27	3	30	133 (0.186)
UWOPS05-227-2	MA	Structured	Structured clean	24	7	31	70 (0.108)
YS4	OM	Mosaic	Mosaic	9	24	33	88 (0.110)
DBVPG 1853	OM	Mosaic	Mosaic	14	23	37	144 (0.205)
Total				339	315	654	

Notes: Table of SNP and pSNP polymorphisms for each *S. cerevisiae* strain, compared to the reference strain S288c, as identified using the TURNIP software. Polymorphism counts are taken from West et al. (in preparation). For each strain, the strain group (geographic or phylogenetic origin/industrial usage), the genome type (mosaic or structured), the modified genome type (mosaic, structured clean, and structure mosaic) determined in this study, and the estimated ribosomal DNA copy number (along with the standard error of the copy number estimate) are also given. Key for groups: MA (Malaysian); NA (North American); SA (Sake); WA + (West African + other mosaics); W/E (Wine/European); YII (strain YIIc17-E5); UM (UWOPS mosaics); OM (Other Mosaics)

### Phylogenetic Tree Estimation and Analysis

Intraspecies phylogenetic trees were estimated, rooted with the chosen strain from the other species. For the *S. paradoxus* tree, the TURNIP output of the 26 *S. paradoxus* strains plus S288c *S. cerevisiae* type strain compared with the rDNA consensus sequence of CBS432 was processed using a custom Perl script (available at NCYC) to construct a matrix containing the frequencies of each nucleotide base in each strain, for all sites with pSNP/SNP polymorphisms. The resulting frequency matrix was then used as input to selected programs within the Phylip phylogenetic analysis suite (Felsenstein 2004, version 3.69). Specifically, a distance matrix was produced using GENDIST with the Cavalli-Sforza and Edwards Chord distance (Cavalli-Sforza and Edwards 1967). A neighbor-joining tree (Saitou and Nei 1987) was generated from this matrix using the NEIGHBOR program. A total of 1000 bootstrap datasets (Felsenstein 1985) were produced from the variation output using SEQBOOT and were subsequently analyzed using GENDIST and NEIGHBOR. The bootstrap trees were mapped to the original tree using RAxML (Stamatakis 2006, version 7.3.0) and the resulting bootstrapped *S. paradoxus* tree was visualized using MEGA 5 (Tamura et al. 2011). These steps were repeated for the *S. cerevisiae* dataset. NeighborNets for both datasets were produced with SplitsTree4 (Huson and Bryant 2006, version 4.12.3), using the Cavalli-Sforza and Edwards Chord distance matrices as input.

The estimated trees were compared with phylogenies previously constructed from genome-wide SNP variation (Liti et al. 2009). *Saccharomyces paradoxus* and *S. cerevisiae* distance matrices, derived from 623 287 and 235 127 nuclear genome SNPs respectively, were downloaded from the SGRP website and were analyzed using NEIGHBOR with strains additional to this analysis removed using RETREE. Subsequent tree comparison was carried out using the software TOPD/FMTS (Puigbò et al. 2007) with the *disagree* option. The value of the resulting Split Distance statistic was compared to those calculated for 100 trees of the same strain set randomly generated by TOPD/FMTS. For each species, the correlation between the rDNA- and SNP-based distance matrices was assessed using the Mantel test within the QIIME software (Caporaso et al. 2010).

### Copy Number Estimation

In our recent analysis (West et al., in preparation), we calculated the coverage of the SGRP sequence reads across the rDNA unit for each strain, using a custom Perl script (available at NCYC and from the Dryad data repository http://dx.doi.org/10.5061/dryad.0674n) to count the number of reads which were hits in each 20-bp window along the rDNA reference unit. Here, we used those results to calculate the average number of sequence reads across the whole rDNA unit for each strain, dividing this number by the calculated average genome coverage for the relevant strain as given in the SGRP user manual (SGRP), to provide an estimate of copy number (Tables 1 and 2). Standard errors of the copy number estimates were calculated, treating genome coverage as a constant.

## Results

### rDNA-based Phylogenetic Analysis of *S. paradoxus* Strains

**Figure 1.**
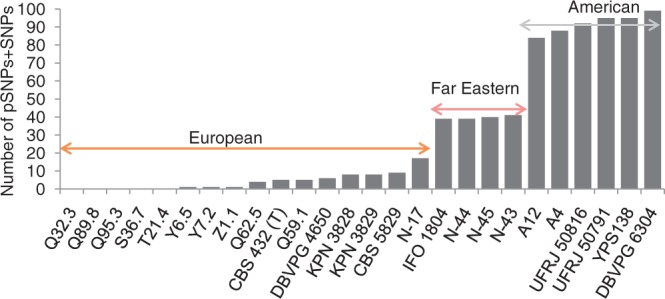
pSNP + SNP polymorphism counts in *S. paradoxus* strains. Bar chart of pSNP plus SNP variation in each *S. paradoxus* strain, labeled to show the split into distinct populations. The strains are ordered by increasing number of pSNPs + SNPs, and naturally split into the three geographical locations.

**Figure 2.**
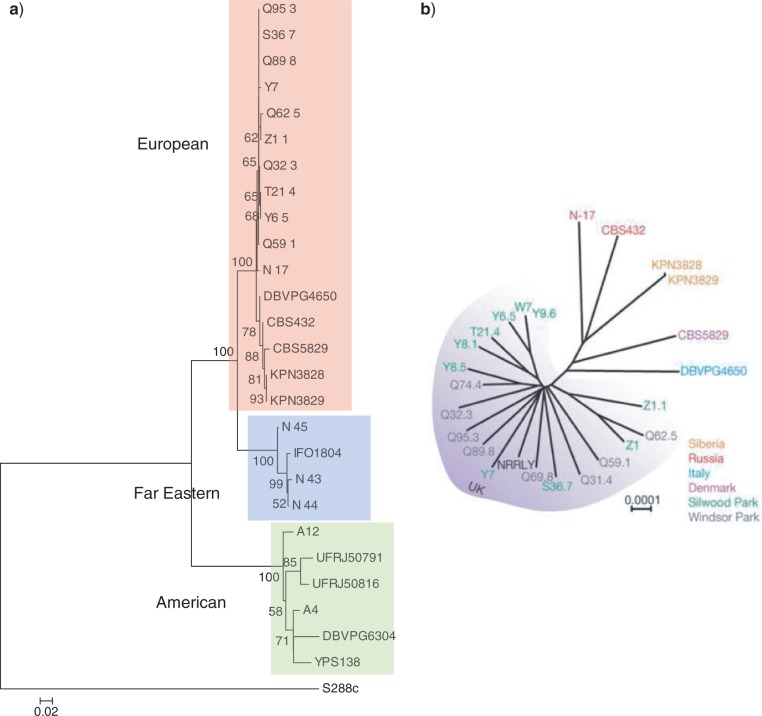
Neighbor-joining phylogenetic trees of the *S. paradoxus* strain set. a) *Saccharomyces paradoxus* neighbor-joining phylogenetic tree with *S. cerevisiae* strain S288c as the nominated root. Bootstrap support values greater than 50 shown. Clear separation into strain collection site can be observed. Little variation within the European group, particularly the 10 UK strains (Q95.3 to Q59.1) and the 2 Siberian strains (KPN3828 and KPN3829), is apparent. N-45 is found to be the most divergent of the 4 Far Eastern strains. The American strains proved to be most divergent as a group. b) *Saccharomyces paradoxus* phylogenetic tree derived from 623 287 genome-wide SNPs (Liti et al. 2009). Reprinted by permission from Macmillan Publishers Ltd: Nature 458:337-341, ©2009.

The phylogenetic signal in the dataset appeared to be strong, with raw pSNP + SNP polymorphism counts highly correlated to geographical origin (American, European, and Far Eastern; Pearson's *r* = 0.987) (Table 1, Fig. 1). The SNP and pSNP polymorphisms identified in the rDNA arrays of each of the 26 *S. paradoxus* strains plus *S. cerevisiae* strain S288c, with the rDNA consensus sequence of *S. paradoxus* strain CBS432 as a reference, were closely examined. Differences between S288c reads and the CBS432 reference sequence, which were not shared with *S. paradoxus* strains, were found in the form of 244 SNPs. SNPs and pSNPs were found to occur between *S. paradoxus* strains at 151 and 58 rDNA sites respectively, at 167 unique positions. Due to the significant overlap of these two variation types, they were combined into a single data matrix with allele frequencies for the 411 polymorphic sites recorded for each strain (online Appendix 1). For each of the 109 sites coded only as *S. paradoxus* SNPs and the 244 S288c SNPs, all entries in the corresponding column would of course be either 0 or 1 but for the 42 coincident SNP/pSNP and 16 pSNP-only sites, entries could take any value between 0 and 1. The resulting rDNA-based phylogenetic tree (Fig. 2a), estimated from the pSNP/SNP allele frequency matrix mirrored the pattern observed in Figure 1, splitting into three well-supported groups that directly corresponded to geographical origins.

A manual inspection of pSNP and SNP variation across strains (Table 3), using the *S. paradoxus* phylogeny as a framework on which to view it, showed a fairly small number of phylogenetic patterns with regard to the within-*S. paradoxus* polymorphisms. The backbone of the tree could be seen to be derived from the 109 inter-genomic SNPs, upon which the remainder of the variation further elaborated. In all, 103 SNPs occurred only within the American group, 15 of which were strain-specific (8 in DBVPG6304 and 7 in YPS138). Only 2 SNPs occured in the Far Eastern group and 3 occurred in the European group, with each group possessing one single-strain SNP each. The only SNP to affect strains in more than one geographical group was observed in the 4 Far Eastern strains and a single European strain (Q62.5). In all, 15 of the 16 pSNP-only polymorphisms were observed in just a single strain, 10 within European strains (6 in N-17), 4 within Far Eastern strains (2 in N-45) and 1 within an American strain (DBVPG6304). Only 1 pSNP was shared across geographical groups, observed in 2 European strains (N-17 and Q59.1) and 1 Far Eastern strain (N-45). The 42 shared pSNP/SNP sites were more difficult to categorize, containing mixtures of SNPs and pSNPs across groups (though never more than two strains containing pSNPs per site), in six cases across all three. However, some patterns stood out, notably the 33 high-frequency pSNPs in the Far Eastern strain N-45, 10 of them coincident with low-frequency pSNPs in the European strain N-17. In 11 cases, the CBS432 (type strain) nucleotide appeared to be a derived state (i.e., a European SNP), with Far Eastern and American group strains possessing the ancestral state.

**Table 3.** Phylogenetic grouping of polymorphisms in *S. paradoxus* and *S. cerevisiae*

Polymorphism type		*S. paradoxus*	*S. cerevisiae*
SNPs	Within strain	17	18
	Within group	91	11
	Across group	1	3
	With root	244	291
pSNPs	Within strain	15	66
	Within group	0	22
	Across group	1	7
pSNPs + SNPs	Within group	16	14
	Across group	26	36
	Total	411	468

Notes: The number of polymorphisms of each type (SNP, pSNP, or pSNP + SNP) across the entire strain set for *S. paradoxus* and *S. cerevisiae*.

Notably, our new rDNA-based phylogeny was highly similar to that previously produced by Liti et al. (2009) (Fig. 2b), generated from 623 287 SNPs spread across the nuclear genome. The grouping of strains into European, Far Eastern and American, and furthermore into UK and non-UK within the European group, was identical between the two trees. Minor differences in topology were seen within-group, with N-17, CBS432, N-45 and A12 the clearest examples. Comparing the two trees using the TOPD/FMTS software (Puigbò et al. 2007), the disagree statistic exhibited a Split Distance of 0.52 compared with a random Split Distance (using randomly generated topologies of the same strain set) of 0.99, reinforcing the closeness of the two phylogenies. This result was further supported by the Mantel test assessing the correlation of the rDNA- and SNP-based distance matrices, with *r* = 0.99029 (*P* = 0.001).

### rDNA-based Phylogenetic Analysis of *S. cerevisiae* Strains

**Figure 3.**
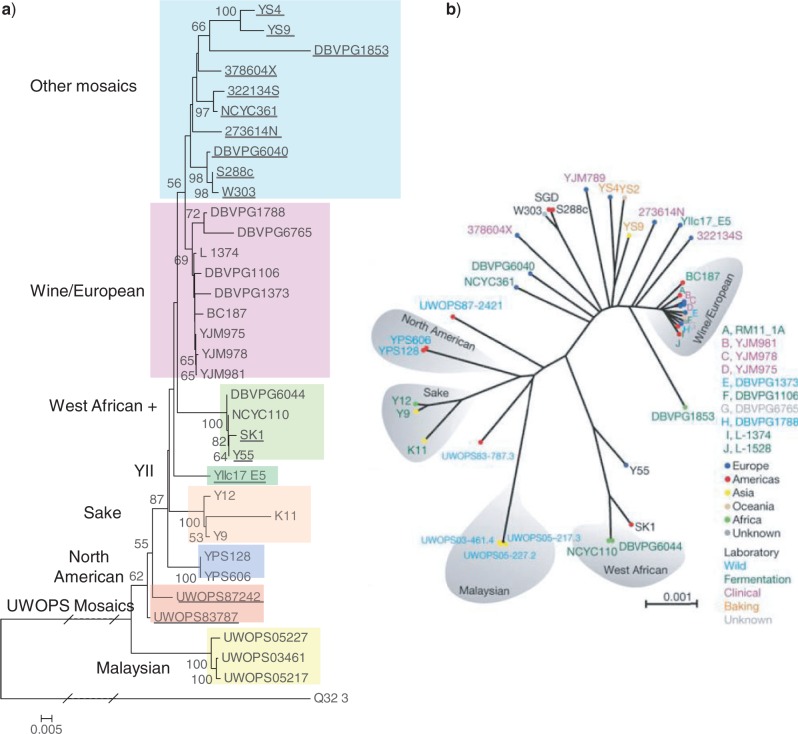
Neighbor-joining phylogenetic trees of the *S. cerevisiae* strain set. a) *Saccharomyces cerevisiae* neighbor-joining tree with *S. paradoxus* strain Q32.3 as the nominated root. Bootstrap support values greater than 50 shown. Dotted line equivalent to 0.355 units of distance. Groups of interest are shown as colored boxes. b) *Saccharomyces cerevisiae* phylogenetic tree derived from 235 127 genome-wide SNPs (Liti et al. 2009). Reprinted by permission from Macmillan Publishers Ltd: Nature 458:337-341, ©2009.

In our new analysis of 34 *S. cerevisiae* strains and *S. paradoxus* strain Q32.3, with the rDNA consensus sequence of *S. cerevisiae* strain S288c as a reference, within-*S. cerevisiae* SNPs and pSNPs were found to occur at 82 and 145 rDNA sites, respectively, at 177 unique positions. An additional 291 SNPs were found between reads of *S. paradoxus* strain Q32.3 and the S288c consensus sequence. As before, an allele frequency matrix was constructed for the combined dataset (online Appendix 2) and a phylogenetic tree was estimated (Fig. 3a). Superimposing the pSNP and SNP variation onto this new *S. cerevisiae* phylogenetic tree (and considering the eight strain groupings in Table 2) showed a greater number of phylogenetic patterns than the *S. paradoxus* dataset (Table 3). Only 32 polymorphisms were found to occur only as within-*S. cerevisiae* SNPs in this dataset. Of those, 18 were found to be strain-specific (including 8 in K11, 5 in DBVPG1853 and 3 in DBVPG6765). Of the remaining 14 SNPs, all of which occurred in between 2 and 4 strains, six were shared by all strains in the WA + group, 3 by both strains in the NA group and 2 by all strains in the MA group. Only 3 SNPs were found in strains belonging to two different groups. In contrast, 95 polymorphisms were found to occur just as pSNPs, 66 of which were strain-specific. The single strain pSNPs were identified in 24 strains spanning all groups except MA, 16 of them in strain DBVPG1853 and 6 in strain DBVPG1373. A further 22 pSNPS, found to occur in between two and six strains, belonged only to a single group (16 to group OM, 3 to W/E, 2 to SA, and 1 to WA + ). Only seven pSNPs were found in strains spanning two or three groups, five of them involving the YIIc17-E5 strain. Fifty polymorphisms were found to occur both as pSNPs and SNPs in the *S. cerevisiae* dataset, with each one involving between 2 and 24 strains. Unlike the *S. paradoxus* dataset, where a maximum of two strains possessed such a pSNP, in *S. cerevisiae* this could involve as many as 12 strains carrying a single pSNP in different occupancy frequencies in addition to other strains where they were fully resolved as SNPs. Fourteen pSNP + SNP polymorphisms occurred in a single group, ten of them in OM and four in SA. The remaining 36 polymorphisms involved between two and four groups, 10 of them across the basal MA and UM groups, and 6 across MA, UM, and OM. As before, we detected several sites (in this case eight) at which the S288c (reference strain) nucleotide appeared to be a derived state.

In a previous *S. cerevisiae* phylogeny based on 235 127 genome-wide SNPs (Liti et al. 2009) (Fig. 3b), 19 *structured* strains were shown to group according to either geographic origin or industrial usage, with the remaining 15 *mosaic* strains more loosely clustered within the tree. Our new rDNA-based phylogeny (Fig. 3a) is highly similar to the whole-genome SNP tree. For example, our new tree exhibits identical Malaysian, North American, Sake, West-African, and Wine/European groups (all consisting of structured strains). Furthermore, there is an overall consistency in the relationships between the groups. The major differences between the two topologies are the more basal position of the Wine/European group in the new rDNA tree and the location of the YIIc17-E5 strain. The varying location of this mosaic strain between the two trees could be explained by its putative parentage, with different relative contributions of its parents within the genome-wide SNP and rDNA datasets. Indeed, on closer examination of the YIIc17-E5 pSNP/SNP polymorphisms, of the 25 rDNA sites at which this strain varies from the reference strain, two contrasting phylogenetic signals can be observed. One set of polymorphisms links YIIc17-E5 to the three Sake strains, whereas the other set links it to the OM group, in particular the 273614N, DBVPG6040, and S288c strains. Comparing the two trees using the TOPD/FMTS software (Puigbò et al. 2007), the disagree statistic exhibited a Split Distance of 0.65 compared to a random Split Distance of 0.99. Furthermore, the Mantel test performed on the two distance matrices supported a strong correlation between them, with *r* = 0.64133 (*P* = 0.001). Although the two trees are not as close as the *S. paradoxus* trees, these results support our observation that there is strong agreement between them.

### NeighborNet Analysis

Online Appendix 3 shows NeighborNets (Bryant and Moulton 2004) estimated for the *S. paradoxus* and *S. cerevisiae* strain sets. It is clear from these networks that the phylogenetic signal exhibited by the *S. cerevisiae* dataset is less tree-like than that of the *S. paradoxus* dataset. In the latter, the three geographical groups are cleanly separated on the network, with the most obvious phylogenetic conflicts occurring within-group, notably involving the American strain UFRJ50791 and the Far Eastern strain N-45. The structured *S. cerevisiae* strains tend to be, with respect to one another, closely grouped with only small incompatible splits (box-like structures) relating them, the major exception being the W/E strain DBVPG6765. In contrast, the mosaic strains show a greater number and size of incompatible splits relating them, particularly within the OM group to the right of the network. Notably, the location of the YIIc17-E5 strain falls between the SA and OM groups in the *S. cerevisiae* NeighborNet, indicating its likely hybrid origin.

Examining the cross-group variation in the two datasets (Table 3) makes clear the reasons for the observed differences between the NeighborNets. Although the *S. paradoxus* dataset possesses several cross-group polymorphisms (e.g., 26 for the pSNP + SNP class), and even though almost all of these cases involve shared polymorphisms at nonadjacent regions of the phylogeny, the majority are cases where one or two pSNPs are shared at low occupancy (i.e., within only a small proportion of sequence reads covering the rDNA site in question) with pSNPs/SNPs found in a single different phylogenetic region. Consequently, the non–treelike signal is still relatively low. In contrast, in *S. cerevisiae* shared polymorphisms are found in a greater number of nonadjacent regions (up to four). Furthermore, because more pSNPs in this dataset are found at medium or high occupancy, where a non–treelike signal exists it tends to be stronger than for the low occupancy pSNPs more prevalent in *S. paradoxus* (see below). Together, these factors account for the less treelike *S. cerevisiae* NeighborNet.

### Partial Single Nucleotide Polymorphisms

We have previously shown pSNP number to be an indicator of genome type in *S. cerevisiae* (James et al. 2009). In our recent study, we identified 73 pSNPs in the *S. paradoxus* dataset, an average of 2.81 pSNPs per strain (Table 1; West et al., in preparation). Over half the strains (15/26 strains) were found to have no pSNPs in their rDNA arrays, with the remainder possessing between 1 and 36 pSNPs. The majority of *S. paradoxus* pSNPs (72.6%) were detected in just two strains, namely N-17 (European strain; 23.3%) and N-45 (Far Eastern strain; 49.3%). Furthermore, most pSNPs occurred at either low ( < 10%) or high ( > 90%) occupancy, with only five pSNPs falling between these two values. In contrast, 315 pSNPs were detected in the *S. cerevisiae* dataset, an average of 9.26 per strain (Table 2). In addition to a much higher pSNP frequency per strain than for *S. paradoxus*, this type of mutation was more evenly spread across the *S. cerevisiae* strains and occurred at a wider range of occupancy values, with 59.7% of the 315 pSNPs possessing an occupancy of between 10% and 90%.

### rDNA Copy Number

**Figure 4.**
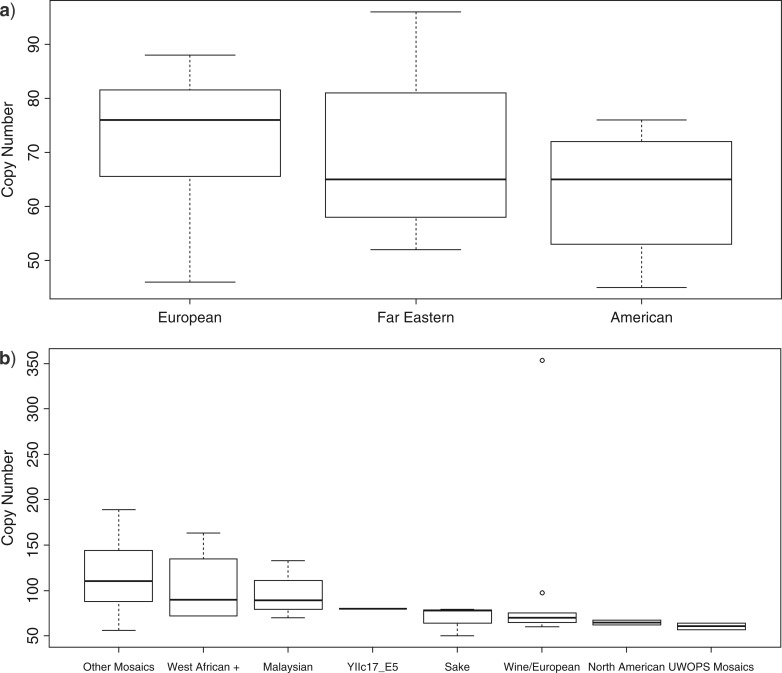
The variation of copy number within groups. a) Box plot of *S. paradoxus* geographical groups and copy number of each strain. b) box plot of *S. cerevisiae* groups versus copy number of each strain. Outliers YJM981 and DBVPG 1106 are represented as circles in the Wine/European group.

The number of rDNA repeats (copy number) in each *S. paradoxus* strain was estimated by comparing the coverage of the rDNA repeat consensus unit to the coverage of the whole genome. The copy number was estimated to range from 45 (American strain A12) to 96 copies (Far Eastern strain IFO1804) (Table 1), with an average of 69 copies per strain. These estimates were found to be lower and less variable than for *S. cerevisiae*, where estimated rDNA copy number ranged from 50 (Sake strain K11) to 354 copies (Wine/European strain YJM981) (Table 2), with an average of 99 copies per strain. Correlation and regression analyses (Table 4) showed there to be no significant association between rDNA copy number and geographical origin in *S. paradoxus* (Fig. 4a). However, in *S. cerevisiae*, once two outliers were removed (YJM981 and DBVPG1106, both Wine/European strains with 354 and 98 copies, respectively) a correlation between copy number and geographical/industrial strain group and between copy number and genome type (and modified genome type, see “Discussion” Section) could be seen (Table 4; Fig. 4b). A strong relationship therefore exists between phylogenetic grouping and rDNA copy number in *S. cerevisiae*, but not in *S. paradoxus*. It would be interesting to determine the factors driving copy number evolution in future studies.

**Table 4.** Correlation and regression analysis of rDNA copy number with strain features

Species	Factor 1	No. of strains	Factor 2	No. of levels	*r*	*P*
*Saccharomyces paradoxus*	rDNA copy number	26	Geographical origin	3	− 0.287	0.293
*Saccharomyces cerevisiae*	rDNA copy number	34	Strain group	8	− 0.253	0.240
	rDNA copy number	34	Genome type	2	− 0.057	0.676
	rDNA copy number	34	Modified genome type	3	− 0.037	0.896
	rDNA copy number	32	Strain group	8	− 0.627	3.89 × 10^−5^^a^
	rDNA copy number	32	Genome type	2	0.299	0.049
	rDNA copy number	32	Modified genome type	3	− 0.129	0.006

Notes: Pearson's correlation coefficients (*r*) were calculated between rDNA copy number and strain features for various numbers of strains. Negative Binomial Generalised Linear Models were also fitted to the same datasets, with *P*-values for the resulting χ^2^ analysis of deviance test also found.

^a^Furthermore, the Negative Binomial regression indicated that the rDNA copy numbers of the Sake, Wine/European, North American and UWOPS Mosaics groups were significantly different from possessed by the Other Mosaics group, with *P* = 0.002, *P* = 1.09 × 10^−5^, *P* = 0.003, and *P* = 0.001 respectively.

## Discussion

### The Use of rDNA in Phylogenetic Analysis

We have shown, for the first time, how a detailed characterization of interconnected systems of pSNPs and SNPs within the rDNA unit can be coded as allelic variation, and how this variation can be analyzed with existing tools to estimate phylogenetic trees (Figs. 2a and 3a) that are highly similar to those estimated in previous genome-wide SNP analyses (Liti et al. 2009). For *S. paradoxus*, this is perhaps not so unexpected, as the majority of pSNPs either have a high occupancy (over 90%) where they will be treated similarly to SNPs or low occupancy (less than 10%), where they will not contribute significantly to pairwise distances. However, this does tell us that the evolutionary pattern within the rDNA region, which changes via concerted evolutionary mechanisms, is highly similar to that across the nuclear genome, which evolves via rather different processes. Furthermore, we also see good agreement between our new rDNA-based phylogeny and previously estimated trees for *S. cerevisiae*, where occupancy ranges are much different and network-like signals resulting from hybridization events are more prevalent. An association between rDNA copy number and *S. cerevisiae* phylogenetic clades was also observed for the first time, but this was not repeated for *S. paradoxus*, an observation that requires a deeper investigation of these and other rDNA datasets.

Interestingly a recent computational study of SGRP *S. cerevisiae* genomes, plus additional genome sequences from SGD (SGD) for validation, showed that a minimal set of 13 specific genes can capture the phylogenetic relationship inherent to these strains (Ramazzotti et al. 2012). The method was proposed as a simpler alternative to whole-genome sequencing, and is highly attractive when financial or analytical constraints are a factor. However, some major challenges were faced by this approach, in particular the inconsistency of gene content across strains. Conversely, our analysis has shown that a single, complex locus may satisfy many of the goals of this study while also being universal across and within species. However, developing datasets such as we are using here would be unachievable for many at the present time. It would be interesting to see whether future technologies could achieve full sequence characterization of the rDNA locus without the need for whole-genome sequencing.

Perhaps uniquely, the rDNA unit offers the opportunity to capture intragenomic sequence variation before it is fixed as a SNP (or conversely is lost), and therefore is ideal for understanding the relationships between members within a species, such as we have analyzed here. In future, it would be interesting to test formally whether pSNPs within the rDNA array (or indeed from other repetitive genomic sequences known to be moulded by concerted evolutionary processes) have greater power than SNPs to discriminate between organisms within species.

### pSNPs as a Predictor of Genomic Mosaicism

In a previous study (James et al. 2009), we showed that a high pSNP count was observed in *S. cerevisiae* strains possessing mosaic genomes (i.e., resulting from a hybridization event). In our present study, we found that on average *S. cerevisiae* strains have 3.25 times more pSNPs in their rDNA arrays than *S. paradoxus* strains (compared to 2.9 in our previous study), with the 15 *S. cerevisiae* mosaic strains possessing 4.44 times more pSNPs than the *S. cerevisiae* structured strains. Statistical tests (Pearson's *r* = 0.713; Negative Binomial regression *P* = 5.15 × 10^−9^) further supported a link between pSNP count and genome type (i.e., mosaic or structured) for this species.

We further identified potential mosaic-like features in *S. cerevisiae* lineages previously categorized as “clean.” Based on pSNP occupancy, the five *S. cerevisiae* structured lineages identified by Liti *et al.* (2009) can be subdivided into two groups, which we subsequently refer to as *structured mosaic* and *structured clean* strains (online Appendix 4, Table 2). In the original set of 15 *S. cerevisiae* mosaic strains, ∼60% of the detected pSNPs (145/245) were found to have occupancies greater than 10% but less than 90%. One scenario under which this type of pSNP could have arisen is if two parental strains from different populations/lineages, and with differing SNPs, crossed and produced a hybrid. Using the mid-occupancy class of pSNP as an indicator of genome mosaicism, we observe that the seven strains belonging to the Malaysian, North American, and West African lineages only have two (out of 16) pSNPs that have occupancies between 10% and 90%, classifying them as structured clean strains. In contrast, the majority of pSNPs (40 out of 54) in the 12 strains belonging to the Sake and Wine/European lineages have occupancies within the 10% to 90% range, showing mosaic-like behavior and classifying them as structured mosaic strains (online Appendix 4). This classification is supported for many of these strains by a reexamination of the Structure diagrams produced by Liti et al. (2009), suggesting the possibility that this class of pSNP might prove useful as a potential indicator of cryptic genome mosaicism, perhaps the result of hybridization events older than those leading to the standard mosaic class. As many of the structured mosaic strains have a fermentation origin (e.g., sake and wine), it is likely they have undergone some degree of hybridization during their respective histories which has left a residual signal within their genomes, including within their rDNA arrays.

The majority of *S. paradoxus* strains show no strong evidence of mosaicism when examining pSNP counts. In a previous study, Liti et al. (2009) identified only one candidate *S. paradoxus* strain (not examined here) as having a potential mosaic-like genome. However, the European strain N-17 (from Russia) and the Far Eastern strain N-45 (also isolated in Russia, albeit in the eastern region of the country) are atypical of *S. paradoxus* strains in that they possess high numbers of pSNPs (Table 1), collectively totalling 72.6% of all pSNPs in this dataset. Both N-17 and N-45 possess low numbers of rDNA units sharing polymorphisms with the Far Eastern and European groups, respectively (and indeed on 11 occasions within shared pSNP-only or pSNP + SNP polymorphisms). This indicates a potential European/Far Eastern hybrid origin for both strains, but with contrasting proportions of these two lineages within their genomes. Three Far Eastern and six European strains were isolated either from oak tree bark or exudate on the same continental land mass. The existence of a region in mainland Europe (perhaps Russia) where European and Far Eastern strains coexist is therefore a possibility, with such a region a potential source of hybrid strains. Although further research would be needed to confirm the N-17 and N-45 hybridizations, the potential to identify hybridization signals from features of rDNA polymorphisms, in organisms with population structures similar to *S. paradoxus*, is intriguing.

Examination of the NeighborNet for *S. paradoxus* (online Appendix 3a) shows a clear phylogenetic conflict implicating the American strain UFRJ5079. Further examination of the source of this conflict shows that it derives from incompatible sharing of SNPs between different subsets of strains within the American group, with one explanation being a recent intragroup hybridization. It is interesting to contemplate the clarity of this SNP-based conflict with our two putative pSNP-based mosaics, which are more difficult to pinpoint on the NeighborNet. Further research could be carried out to determine whether pSNP-based conflicts can be easily identified using such tools or whether this is simply a consequence of potentially old events exhibiting low-frequency pSNPs in this particular case.

The combination of whole-genome SNP analysis and rDNA analysis, particularly in the case of *S. cerevisiae*, has enabled us to observe links between genome mosaicism predicted by sequence recombination/STRUCTURE analysis and pSNP number/frequency respectively. Furthermore, the NeighborNet derived for this species, largely descriptive of this mosaicism, is itself a product of homoplasy within the rDNA dataset, in particular the across-group categories of polymorphisms noted in Table 3. We note that homoplasy was one of the criticisms of the use of ITS sequences in plant phylogenetic analysis. Indeed, genome evolution in plants has many characteristics in common with yeasts, such as frequent genome hybridization. Consequently we speculate that, in some cases at least, homoplasy will in fact derive from genome mosaicism. In such cases, a detailed examination of pSNPs could provide additional information on the origins of the genomes undergoing analysis.

## Conclusions

We have shown that sequence variation present within the rDNA locus, when characterized in fine detail, can be transformed from a phylogenetic problem to a rich source of evolutionary information from which accurate phylogenetic reconstruction may be achieved. Furthermore, we have refined our previous association between pSNP counts and genome type. For species where hybridization is relatively frequent, such as for *S. cerevisiae*, pSNP occupancy can provide additional information regarding genome structure. Conversely, where hybridization is infrequent, coincident with a lower prevalence of pSNPs, pSNP counts may still provide an indication of rare, putative hybridization events. Given the strong connection between the rDNA locus and phylogenetic estimation, we believe this new knowledge could be useful for many researchers, particularly those working within a species group.

Although we have shown that phylogenetic analysis of rDNA micro-heterogenity datasets is relatively straightforward for the yeast species examined here, which possess a single rDNA locus, we must consider the many organisms that possess multilocus systems. A sequence-based approach such as this could be adapted simply to a multilocus case, particularly where the different loci are considered distinct (although more complex schemes are also possible). This would almost certainly require prior knowledge of genome organization, in order to distinguish between the various paralogous loci, so would not initially be suitable for many species for which this information is not available or where sequenced reads may not be obtained due to lack of facilities or funds. However, with the advent of inexpensive sequence data more rapidly and widely available, we believe that such analyses could become routine within but a few years, providing rapid phylogenetic estimation without the need for whole-genome characterization, at present a highly time-consuming process. We will shortly begin a detailed analysis of rDNA sequence variation in multilocus organisms, beginning with bilocus yeast species. We aim to formally extend our approach, further enhancing the rDNA locus as an information-rich phylogenetic marker.

## Supplementary Material

Data available from the Dryad Digital Repository: http://dx.doi.org/10.5061/dryad.0674n.

## Funding

C.W. was supported by a Doctoral Training Program grant from the Biotechnology and Biological Sciences Research Council (BBSRC). S.A.J., R.P.D., J.D., and I.N.R. all received BBSRC support via their respective institutes. The NCYC is a BBSRC supported National Capability.

## Appendix 1

### Saccharomyces paradoxus Variation Table

File type: xlsx

pSNP and SNP frequencies for SGRP sequence reads of 26 *S. paradoxus* strains and the S288c *S. cerevisiae* strain compared with the rDNA consensus sequence of the CBS432 *S. paradoxus* type strain.

## Appendix 2

### Saccharomyces cerevisiae Variation Table

File type: xlsx

pSNP and SNP frequencies for SGRP sequence reads of 34 *S. cerevisiae* strains and the Q32.3 *S. paradoxus* strain compared with the rDNA consensus sequence of the S288c *S. cerevisiae* type strain.

## Appendix 3

### Phylogenetic Networks of S. paradoxus and S. cerevisiae Strains

File type: svg

Both a) and b) show an enlargement of the main population structure in the network, with the small gray inset showing the whole network including the outgroup. a) The *S. paradoxus* network shows a clear separation of each geographic population. b) The *S. cerevisiae* network shows a more complex network structure, consistent with our knowledge of this population.

## Appendix 4

### Bar Charts of the pSNP Percentage Occupancy in S. cerevisiae by Population Type

File type: pdf

a) Bar chart of the *S. cerevisiae* structured strains, with number of pSNPs against the pSNP occupancy. The boxed section highlights pSNPs with occupancies greater than 10% and less than 90%. The Malaysian, North American, and West African strains have very few pSNPs within this boxed area, and these are denoted as structured clean strains. Those strains with a number of pSNPs within this boxed area show a degree of mosaicism, and we classify these strains as being structured mosaic strains.

b) Bar chart of *S. cerevisiae* mosaic strains, where there are a large number of pSNPs within the 10% to 90% occupancy range.
